# Concordance in pathogen identification at the upper and lower respiratory tract of children with severe pneumonia

**DOI:** 10.1186/s12879-023-08127-w

**Published:** 2023-03-20

**Authors:** Heping Wang, Xiaonan Li, Yuejie Zheng, Lilly M. Verhagen, Jiali Gu, Li Li, Zhi Xu, Wenjian Wang, Marien I. de Jonge

**Affiliations:** 1 Shenzhen Children’s Hospital, No. 7019 Yitian Road, Futian District, Shenzhen, Guangdong 518038 China; 2 Department of Laboratory Medicine, Laboratory of Medical Immunology, Radboud Center for Infectious Diseases, Radboud university medical center, Nijmegen, The Netherlands; 3 Department of Pediatric Infectious Diseases and Immunology, Amalia Children’s Hospital, Radboud University Medical Center, Nijmegen, the Netherlands; 4 Ningbo Health Gene Technologies Co., Ltd, Ningbo, Zhejiang China

**Keywords:** Community acquired pneumonia, Pathogen detection, Concordance, Upper and lower respiratory tract, Children

## Abstract

**Background:**

Nasopharyngeal swabs are taken to determine the causative agent of community acquired pneumonia (CAP), while the reliability of upper respiratory tract sampling as a proxy for lower respiratory tract infections is still unclear.

**Methods:**

Nasopharyngeal (NP) swabs, bronchoalveolar lavage (BAL) fluid samples and clinical data were collected from 153 hospitalized children between 3 months and 14 years of age with severe CAP, enrolled from March to June 2019. Written informed consent for the storage and use of the samples for further studies was obtained from the parents or caregivers. Putative pathogens were detected using a sensitive, high-throughput GeXP-based multiplex PCR and qPCR.

**Results:**

The same bacterial species in paired samples were found in 29 (23.4%) and the same viral species in 52 (27.5%) of the patients. moderate concordance was found for *Mycoplasma pneumoniae* (*ĸ*=0.64), followed by *Haemophilus influenzae* (*ĸ*=0.42). The strongest discordance was observed for human adenovirus and also for *Pseudomonas aeruginosa*, the latter was exclusively detected in BAL samples. In the adenovirus cases strong concordance was associated with high viral loads in the NP swabs.

**Conclusion:**

The variation in concordance in pathogen detection in the upper and lower respiratory tract of children with severe pneumonia is generally high but varies depending on the species. Novel and impactful insights are the concordance between NP and BAL detection for *M. pneumoniae* and *H. influenzae* and the strong correlation between high adenoviral loads in NP swabs and detection in BAL fluid.

## Introduction

Community-acquired pneumonia (CAP) is a common childhood disease and often a reason for hospital admission. CAP remains an important cause of morbidity in the developed world and continues to be associated with high mortality in the developing world ^[[1]–[2]]^. CAP can be caused by many different pathogenic microorganisms, most prominently by viruses and bacteria. Although the diverse clinical and radiological presentations of CAP reflect a variety of responsible microorganisms ^[[3]–[4]]^, a clear etiology is essential for clinical diagnosis and treatment. Adequate antimicrobial therapy has been the most important factor for successful management of CAP caused by bacteria [5–7].

Bronchoalveolar lavage (BAL) has traditionally been considered the ’gold standard’ for the etiological diagnosis of lower respiratory tract infections in children [8–10]. However, BAL sampling is complicated, requires the involvement of specialists, as it is often performed under local anesthesia and conscious sedation, and is therefore only conducted for further evaluation of radiologic imaging when patients are severely ill. Replacing BAL by NP swab sampling would strongly reduce diagnostic complexity, however not much is known about the level of concordance between pathogen detection in the upper and lower respiratory tract in CAP. Few studies have been published describing this comparison, in which sample sizes were often too small, mainly in the studies with pediatric patients. Another limitation of these studies was that the upper and lower respiratory tract was not sampled at the same time. Furthermore, most studies were focused on viral detection only [11–16].

In this study, we compared viral and bacterial pathogen detection in the upper and lower respiratory tracts of children with severe pneumonia, to assess the concordance between concurrent upper and lower respiratory tract testing and to estimate to what extent and for which pathogens NP swab sampling could potentially replace BAL sampling to obtain an etiological diagnosis for pediatric patients with CAP.

## Materials and methods

### Patient enrollment and sample collection

Pediatric patients, admitted to Shenzhen Children’s Hospital from March to June 2019, were diagnosed with severe pneumonia based on fast breathing, chest wall indrawing, wheezing in a calm child or any general danger sign (not able to drink or breastfeed, persistent vomiting, having convulsions, being lethargic or unconscious) accompanied by an increase in temperature and abnormalities consistent with pneumonia determined by respiratory specialists based on chest radiographs or chest computer tomography (CT) scans [16]. Children with comorbidities, such as cerebral palsy, neuromuscular diseases, chronic lung disease, or congenital heart diseases, were excluded from this study. Only children with severe pneumonia inadequately responding to antimicrobial therapy requiring bronchoscopy and lavage to identify the cause of infection were selected for this study. We collected both BAL fluid and NP swab samples if they conformed the inclusion criteria. White blood cell (WBC) count, neutrophil count and C-reactive protein (CRP) levels were measured at the day of hospital admission.

BAL fluid samples were collected as part of the standard clinical care following the institutional standard practice [17], using 0.5-1 ml/kg of normal saline. According to the standard protocol the upper respiratory tract of the patients was treated with topical anesthesia before bronchoscopy. To avoid contamination with bacteria and viruses residing in the upper respiratory tract the bronchoscope was not used for aspiration while entering the airways until it reached the lesion in the lungs. The lavage was repeated at the lesion and the first BAL probe of 20 mL was discarded to prevent contamination.

Nasopharyngeal swabs (cat. No. HCPN305C, Copan Italy) were collected in universal transport medium (UTM) and BAL fluid samples were collected concurrently (i.e. at the same time) from each patient. Clinical and demographic data of the patients enrolled in this study were retrieved from the Shenzhen Children’s Hospital electronic patient dossiers. This study was approved by the Ethical Committee of Shenzhen Children’s Hospital with registration number 2,016,013. Written informed consent for the storage and use of the BAL and NP samples for further studies was obtained from the parents or caregivers before enrollment.

### GeXP-based multiplex PCR and in-house qPCR on nasopharyngeal swabs and BAL fluid samples

Total RNA/DNA was extracted from BAL or swab sample (UTM) as previously described [5]. Plasmid pcDNA3.1 (+) (Thermo Fisher Scientific Co Ltd., Shanghai, China) was used as the internal control. The GeXP-based assay (Genome Lab GeXP Genetic Analysis System) was performed on all BAL fluid and swab samples for 11 different respiratory pathogens: human adenovirus (HAdV), *Chlamydophila pneumoniae*, coronaviruses (229E, OC43, NL63 and HKU1), human metapneumovirus (HMPV), human rhinovirus (HRV), human bocavirus (HBoV), influenza A (H3N2, H1N1, H5N2 and H7N9) and influenza B viruses, *Mycoplasma pneumoniae*, parainfluenza virus (types 1, 2, 3 and 4) and human respiratory syncytial virus (HRSV, RSV A and B) (Health Gene Tech., Ningbo, China). The data was analyzed with the GeXP system Software as previously described [14, 16]. In addition, we simultaneously performed semi-quantitative PCR detection on adenovirus-positive specimens using the TaqMan probe PCR kit for HAdV (Shenzhen Puruikang Biotechnology Co., Ltd., Shenzhen, China) [5].

Bacterial species *Streptococcus pneumoniae, Haemophilus influenzae, Moraxella catarrhalis, Staphylococcus aureus, Klebsiella pneumoniae, Acinetobacter baumannii* and *Pseudomonas aeruginosa* were identified by qPCR as previously described [5, 18].

### Statistical analysis

Discordance was defined as either (1) a NP sample positive for a pathogen with the paired BAL sample being negative for the same pathogen or (2) a NP sample negative for a pathogen with the paired BAL sample being positive for the same pathogen. Positive concordance was defined as both NP and BAL samples being positive for the same pathogen and negative concordance was defined as both NP and BAL paired samples being negative for the same pathogen.

The agreement of the results obtained by the two different sample collection methods (NP and BALF) was assessed with the Cohen’s kappa test for each identified microorganism. The sensitivity, specificity, and predictive values of NPA specimens were calculated from 2 × 2 tables, with confidence intervals calculated using the binominal exact method. Logistic regression models were used to evaluate odds ratios (ORs) for the association between epidemiological factors and detection of viruses/bacteria. Covariates that were included in multivariable models are listed in Table 1. Statistical significance was defined as 2-sided *P* < 0.05. All statistical analyses were conducted using SPSS 19 (SPSS Inc. Chicago, IL, USA).

**Table 1:** Patient characteristics

Characteristics			Number (%) of children requiring a bronchoalveolar lavage (total number = 153)	
Age (months)				
		≤ 36	71(46.4)	
		> 36	82(53.6)	
		Median	42	
Sex				
		Male	81(52.9)	
		Female	72(47.1)	
Co-morbidities				
		Asthma or allergic diseases	21(13.7)	
		Congenital diseases of lung	9(5.9)	
Symptoms				
		Fever	126(82.4)	
		Cough	144(94.1)	
		Wheezing	23(15.0)	
WBC		< 5	17(11.1)	
(10^9^/L)		5–12	101(66.0)	
		> 12	35(22.9)	
Neutrophil (%)		< 50	53(34.6)	
		50–70	57(37.3)	
		> 70	43(28.1)	
CRP (mg/L)		≤ 10	87(56.9)	
		> 10	66(43.1)	
Hospitalization				
		Median length of stay-days	8	
		Mechanical ventilation	5(3.3)	
		Oxygen absorption	57(37.3)	
		Antibiotic treatment	149(97.4)	
		Antiviral treatment	19(12.4)	
		Corticosteroid treatment	84(54.9)	
Bronchoscopy time point				
		Days after admission (median)	3	

## Results

### Patient characteristics

The median age of the 153 enrolled pediatric patients requiring a BAL was 42 months (interquartile range, 12 to 60), and the median length of hospital stay was 8 days (interquartile range, 6 to 10). Male to female ratio of the patients was 1.13:1. All patients had radiologically confirmed pneumonia and antibiotics upon admission were administered in 149 cases. A total of 57 patients (37.3%) required administration of supplemental oxygen and 5 (3.3%) required invasive mechanical ventilation (Table 1). There were 9 patients with tracheomalacia, a history of premature birth or pulmonary dysplasia, which were not reasons to exclude them from the study. Five patients were admitted to the ICU in this study.

### Putative pathogens detection in BAL fluid samples

**Fig. 1:**
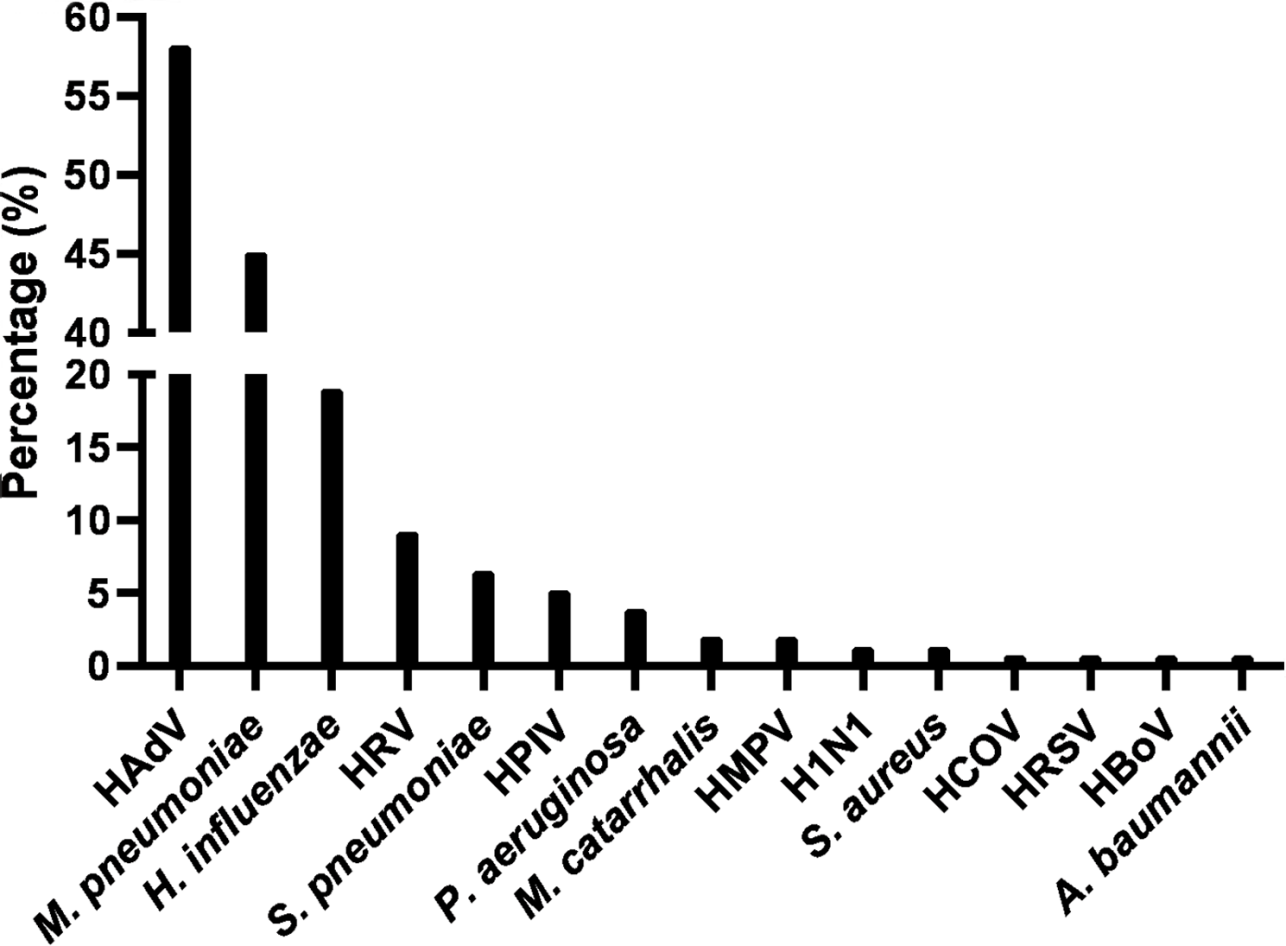
Percentages of viral and bacterial pathogens detected in 153 BAL fluids. HADV, human adenovirus; HRV, human rhinovirus; HPIV, human parainfluenza viruses 1–4; HRSV, human respiratory syncytial virus; HMPV, human metapneumovirus; HCOV, human coronavirus; H1N1, influenza A (H1N1); HBoV, human bocavirus

In 153 BAL fluid samples, we detected one or more pathogens in 143 positive samples (93.5%). Single bacterial or viral pathogens were found in 66 patients (43.1%) while co-infection with multiple pathogens were identified in 77 patients (50.3%). Figure 1 shows the level of putative pathogens in BAL fluid samples. The most frequently detected putative pathogens were HAdV (58.2%), *M. pneumoniae* (45.1%) and *H. influenzae* (19.0%), followed by HRV (9.2%), *S. pneumoniae* (6.5%), HPIV (5.2%), *P. aeruginosa* (3.9%), *M. catarrhalis* (2.0%), HMPV (2.0%), influenza virus H1N1 (1.3%), S. *aureus* (1.3%) and HCOV (0.7%). HRSV (0.7%), HBoV (0.7%), *A. baumannii* (0.7%).

### Putative pathogens detection in NP swabs

**Fig. 2:**
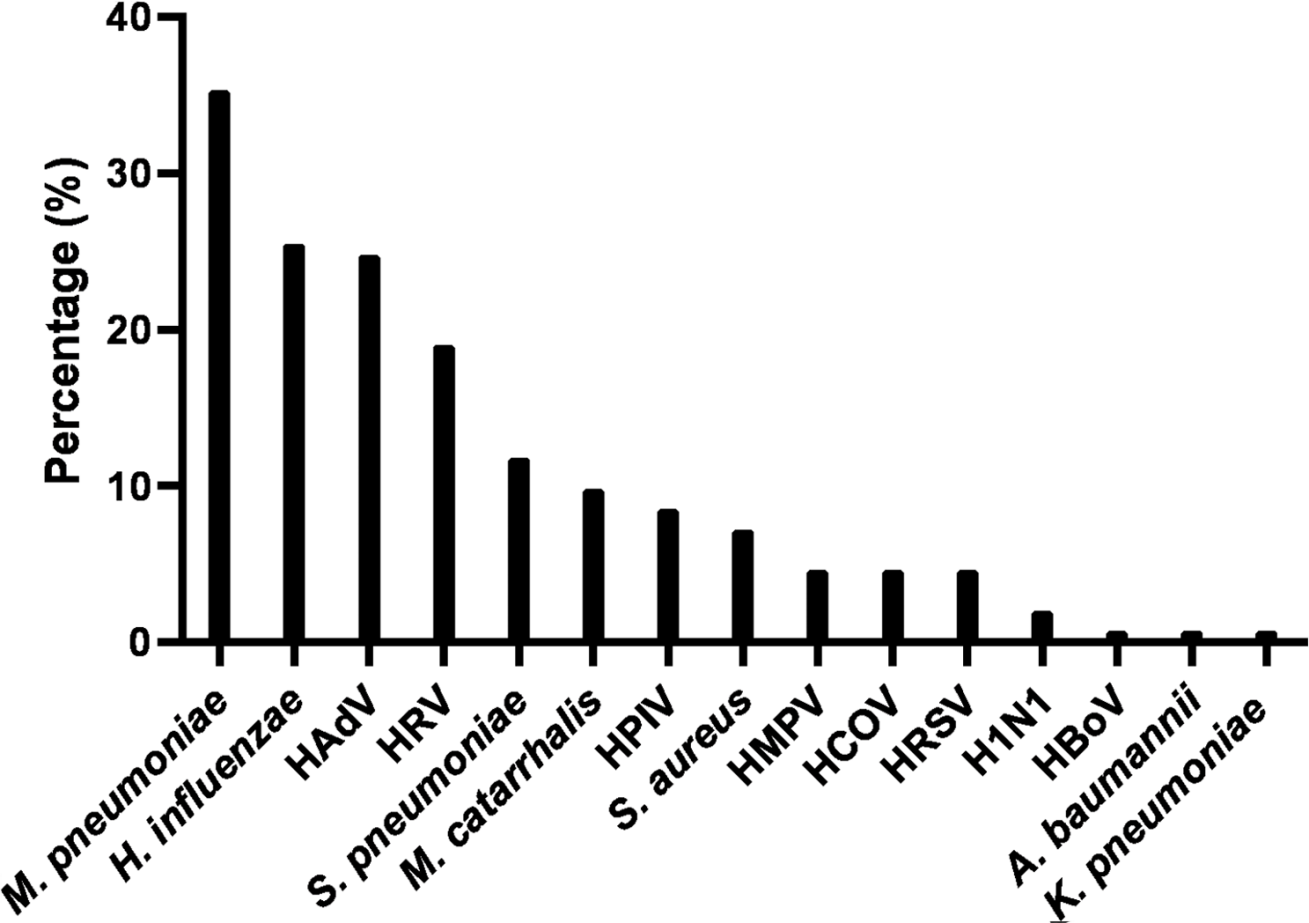
Percentages of viral and bacterial pathogens detected in 153 NP. HADV, human adenovirus; HRV, human rhinovirus; HPIV, human parainfluenza viruses 1–4; HMPV, human metapneumovirus; HCOV, human coronavirus; HRSV, human respiratory syncytial virus; H1N1, influenza A (H1N1); HBoV, human bocavirus

A total of 141 nasopharyngeal swabs (92.2%) were positive for at least one bacterium or virus, and in 47.7% multiple bacteria or viruses were identified. Figure 2 shows the level of putative pathogens in NP swabs. The most common putative pathogens detected by the same PCR-based methods as used for the analyses of the BAL fluids were *M. pneumoniae* (35.3%), *H. influenzae* (25.5%) and HAdV (24.8%), followed by HRV (19.0%), *S. pneumoniae* (11.8%), *M. catarrhalis* (9.8%), HPIV (8.5%), S. *aureus* (7.2%), HMPV (4.6%), HCOV (4.6%). HRSV (4.6%), H1N1 (2.0%), HBoV (0.7%), *A. baumannii* (0.7%), *K. pneumoniae* (0.7%).

### Analysis of paired NP swabs and BAL fluid samples

When considering detection of any potential pathogen, 131 (85.6%) NP-BAL pairs were concordantly PCR-positive, 22 (14.4%) pairs had discordant PCR results and none were concordantly PCR-negative. Generally, respiratory viruses and *M. pneumoniae*, were more often detected in the lower than in the upper respiratory tract samples (35 versus 14 and 21 versus 6, respectively), while respiratory bacteria (except *M. pneumoniae* and *P. aeruginosa*) were most often detected in the upper respiratory tract (38 versus 12).

As low prevalence might strongly influence the positive predictive value, we only assessed the bacterial and viral species that are highly prevalent, i.e. *M. pneumoniae, H. influenzae, S. pneumoniae, M. catarrhalis* and HAdV, HRV, HPIV. Tables 2 and 3 show the level of concordance and discordance for pairs positive for at least 1 pathogen. The strongest discordance, between NP and BAL, was observed for HRV (34 positive pairs, 20 NP+/BAL- and 5 NP-/BAL+), HPIV (17 positive pairs, 9 NP+/BAL- and 4 NP-/BAL+), HAdV (90 positive pairs, 1 NP+/BAL- and 52 NP-/BAL+), *M. catarrhalis* (16 positive pairs, 13 NP+/BAL- and 1 NP-/BAL+), S. *aureus* (12 positive pairs, 10 NP+/BAL- and 1 NP-/BAL+) and *S. pneumoniae* (23 positive pairs, 13 NP+/BAL- and 5 NP-/BAL+).

**Table 2:** The concordance and discordance in specific viral pathogens in NP and BAL samples from 153 patients

NPS/BALF	HADV	HRV	HPIV	HRSV	HMPV	HCOV	INFA/B	HBoV
NPS+	38	29	13	7	7	7	3	1
BALF+	89	14	8	1	3	1	2	1
NPS+/BALF+	37	9	4	0	1	0	1	0
NPS-/BALF+	52	5	4	1	2	1	1	1
NPS+/BALF-	1	20	9	7	6	7	2	1
NPS-/BALF-	63	119	136	145	144	145	149	151
Cohen’s kappa (95% CI)	0.36(0.25–0.47)	0.34(0.14–0.53)	0.34(0.07–0.61)	-	-	-	-	-
X [2]	49.08	7.84	1.23	-	-	-	-	-
*P*	< 0.001	0.005	0.267	-	-	-	-	-

**Table 3:** The concordance and discordance in specific bacterial pathogens in NP and BAL samples from 153 patients

NPS/BALF	*M. pneumoniae*	*H. influenzae*	*S. pneumoniae*	*M. catarrhalis*	*S. aureus*	*P. aeruginosa*	*A. baumannii*	*K. pneumoniae*
NPS+	54	39	18	15	11	0	1	1
BALF+	69	29	10	3	2	6	1	0
NPS+/BALF+	48	20	5	2	1	0	1	0
NPS-/BALF+	21	9	5	1	1	6	0	0
NPS+/BALF-	6	19	13	13	10	0	0	1
NPS-/BALF-	78	105	130	137	141	147	152	152
Cohen’s kappa (95% CI)	0.64(0.52–0.76)	0.47(0.31–0.64)	0.30(0.06–0.54)	0.20(0.05–0.43)	-	-	-	-
X [2]	7.26	2.89	2.72	8.64	-	-	-	-
*P*	0.007	0.089	0.099	0.003	-	-	-	-

*M. pneumoniae* had the highest concordance (Cohen’s kappa = 0.64), followed by *H. influenzae* (Cohen’s kappa = 0.47). *P. aeruginosa* was only detected in BAL fluid samples.

In the case of HAdV detection, cycle thresholds (Ct) values derived from PCR analyses on BAL samples were lower (indicative for a high viral load) than on NP samples, median values were Ct 19.7 and 23.8, respectively. Interestingly. In all concordant HAdV cases, Ct median values in the NP swabs were low (< 20), while in patients where HAdV was only detected in the BAL fluid samples, Ct values were generally higher (Ct median 30.4).

### Identification of associations with increased odds for detection of putative pathogens in the BAL

To further understand which factors are associated with increased odd rates for detection of putative pathogens in the lungs, univariable analyses on the most important covariates were conducted. This showed that age was associated with the detection of viruses in the lungs with a higher rate of detection in older children (> 36 months). Furthermore, NP positivity was identified as an important factor associated with detection of viruses in the lungs. The same covariates were found to be associated with bacterial detection in the lungs, as well as higher CRP levels (> 10 mg/L). In multivariable analyses on all covariates, only detection of viruses and bacteria in NP swab samples were associated with increased adjusted odds for detection in the BAL. Other factors, including age, sex, antibiotic treatment, whole blood counts and CRP levels were not associated with detection of putative pathogens in the lungs (Tables 4 and 5).

**Table 4:** Univariable and multivariable analyses of association for detection of viruses in BAL samples from children with severe pneumonia

Covariates	Categories	Univariable analyses		Multivariable analyses	
		OR (95% CI)	*P* Values	OR (95% CI)	*P Values*
Sex	Female	0.86 (0.43–1.73)	0.68	0.65 (0.30–1.41)	0.28
	Male	1		1	
Age	≤ 36	1		1	
	> 36	2.48 (1.19–5.16)	**0.02**	1.63 (0.68–3.88)	0.27
Antibiotics	Yes	1		1	
	No	1.00 (0.33–3.02)	1.00	1.21 (0.36–4.06)	0.76
WBC on admission day (10^9^/L)	≤ 12	1		1	
	> 12	1.72(0.68–4.32)	0.25	1.34 (0.48–3.78)	0.58
CRP on admission day (mg/L)	≤ 10	1		1	
	> 10	0.59(0.29–1.18)	0.14	0.88 (0.38–2.01)	0.77
Detection of viruses in NP swabs	Negative	1		1	
	Positive	4.00(1.91–8.37)	**< 0.01**	3.47(1.56–7.73)	**< 0.01**

Abbreviations: OR, odds ratio; WBC, white blood cells; CRP, C-reactive protein; NP, nasopharyngeal

**Table 5:** Univariable and multivariable analyses of association for detection of bacteria in the BAL in children with severe pneumonia

Covariates	Categories	Univariable analyses		Multivariable analyses	
		OR (95% CI)	*P* Values	OR (95% CI)	*P Values*
Sex	Female	0.58 (0.29–1.15)	0.12	0.70 (0.33–1.50)	0.36
	Male	1		1	
Age	≤ 36	1		1	
	> 36	0.50 (0.25–0.99)	**0.05**	0.57 (0.25–1.32)	0.19
Antibiotics	Yes	1		1	
	No	1.14 (0.40–3.28)	0.81	0.85 (0.27–2.71)	0.79
WBC on admission day (10^9^/L)	≤ 12	1		1	
	> 12	0.69(0.31–1.52)	0.36	1.08 (0.41–2.81)	0.88
CRP on admission day (mg/L)	≤ 10	1		1	
	> 10	2.79(1.33–5.85)	**0.01**	2.29 (0.98–5.37)	0.06
Detection of bacteria in NP swabs	Negative	1		1	
	Positive	5.19(2.46–10.95)	**< 0.01**	5.08(2.30-11.25)	**< 0.01**

Abbreviations: OR, odds ratio; WBC, white blood cell; CRP, C-reactive protein; NP, nasopharyngeal

## Discussion

In this study, we characterized the rates of concordance for putative respiratory pathogen detection in paired NP and BAL samples taken from patients with severe pneumonia ^[[19]–[20]]^. We found that concordance was largely dependent on the viral and bacterial species. Moderate agreement between detection in NP and BAL was found for *M. pneumoniae* and *H. influenzae*, however poor concordance was found for HAdV, HRV and *S. pneumoniae*. *Pseudomonas aeruginosa* was only detected in the lungs, indicating that this pathogen tends not to colonise the upper respiratory tract in children with community-acquired pneumonia. This has also been found in other studies conducted in pediatric cystic fibrosis patients in which *P. aeruginosa* was found in the lower but not upper respiratory tract ^[[21]–[22]]^. The six children positive for *P. aeruginosa* in our study had either bronchopulmonary dysplasia or severe pneumonia in which adenovirus and *M. pneumoniae* were co-detected. For influenza, a common viral pathogen in children, the detection rate in this study was low, which was due to the season in which the samples were collected. Interestingly, the concordance of detection in NP and BAL samples for this pathogen was high. However, for a reliable conclusion on concordance in detection, paired NP and BAL samples should be collected during the season that influenza detection is high.

Strikingly, for HAdV detection we found that in 100% of the patients with Ct values below 20 measured in NP swab samples, HAdV was detected in the lungs. HAdV is not always appreciated as a significant cause of LRTIs while it can be devastating [23], however this study indicates that if high viral loads are detected in the upper respiratory tract, there is a high chance that HAdV is the causative pathogen in LRTI disease, this is an important finding with significant clinical impact.

Although the concordance of detection of the same virus or bacterium in our study was only 20–30%, in multivariable analysis, positivity of NP swab samples was significantly associated with simultaneous detection of viruses and bacteria in the lungs.

Some of our results were different from previous studies, which is likely due to the differences in patient population. These previous studies were done in chronically diseased and transplanted patients from a different age, mainly adults ^[[8]–[9, 14]]^. Furthermore, the timing of sample collection may have a great impact on the results [14], therefore we collected NP and BAL samples at the same time.

Our study has several limitations. First, although the sample size was larger than previously studies, it might still be too small to draw firm conclusions. Also, the diversity of pathogens was limited. By expanding the sample size, the variety of pathogens may increase thereby improving the relevance of the comparison and the clinical value of the analysis. Secondly, the period in which samples were collected was relatively short, which could also explain the limited number of different pathogens that were identified. This might not reflect the detection of all pathogens in a whole year. Ideally, in pneumonia research studies, the observation period should at least be one year to appreciate seasonality of respiratory pathogens.

Thirdly, both NP swab and BAL sampling methods have limitations. The NP swab is inherently ‘contaminated’ with putative pathogens residing in the upper respiratory tract. However, the BAL, generally considered as the ‘gold standard’, could also potentially be contaminated as a consequence of ‘seeding’, the passage of the bronchoscope through the upper airway.

In conclusion, the level of concordance concerning detection of any pathogen in NP swab and BAL samples is high and positive detection in NP swabs is significantly correlated with higher odds of BAL positivity. The variation in detection rates in the upper versus lower respiratory tract of children with severe pneumonia is generally high but varies depending on the species. Some of the results consolidate previous findings, such as the absence of colonization of the upper respiratory tract by *P. aeruginosa* and the discordance in NP versus lung detection for some viral infections including HRV, known for their asymptomatic occurrence in the upper respiratory tract. More importantly, we discovered moderate concordance between NP and BAL detection for *M. pneumoniae* and *H. influenzae* and a significant correlation between high adenoviral loads in NP swabs and BAL positivity. This will not only improve our understanding of the pathogenesis of respiratory infections but will also support the diagnosis of respiratory tract infections by the improvement of the interpretation of results based on molecular diagnostic analyses on NP swabs.

## Data Availability

All data generated or analyzed during this study are included in this published article.
